# The cost‐effectiveness of radial access percutaneous coronary intervention: A propensity‐score matched analysis of Victorian data

**DOI:** 10.1002/clc.23798

**Published:** 2022-02-22

**Authors:** Peter Lee, Angela Brennan, Diem Dinh, Dion Stub, Jeffrey Lefkovits, Christopher M. Reid, Ella Zomer, Ken Chin, Danny Liew

**Affiliations:** ^1^ Department of Epidemiology and Preventive Medicine, School of Public Health and Preventive Medicine Monash University Melbourne Victoria Australia; ^2^ Cardiology Department Alfred Hospital Melbourne Victoria Australia; ^3^ Cardiology Department Royal Melbourne Hospital Melbourne Victoria Australia; ^4^ School of Public Health Curtin University Perth Western Australia Australia; ^5^ Melbourne Medical School The University of Melbourne Melbourne Victoria Australia

**Keywords:** acute coronary syndrome, cost‐effectiveness, health economics, percutaneous coronary intervention

## Abstract

**Background:**

Despite evidence of the comparative benefits of transradial access percutaneous coronary intervention (PCI) over transfemoral access, its uptake remains highly varied across Australia. Few studies have explored the implications of the choice of access site during PCI from the perspective of the Australian healthcare setting. We, therefore, performed a cost‐effectiveness analysis of radial versus femoral access PCI.

**Methods:**

Data from the Victorian Cardiac Outcomes Registry (VCOR) were used to inform our economic analyses. Patients treated through either radial or femoral access PCI were propensity score‐matched using the inverse probability weighted (IPW) method, and the incidence of major bleeding and all‐cause mortality in the cohort was used to inform an economic model comprising a hypothetical sample of 1000 patients. Costs and utility data were drawn from published sources. The economic evaluation adopted the perspective of the Australian healthcare system.

**Results:**

Among a cohort of 1000 patients over 1 year, there were 19 fewer deaths, and six fewer episodes of nonfatal major bleeding in the radial group compared to the femoral group. Total cost savings attributed to radial access was AUD $1 214 688. Hence, from a health economic point of view, radial access PCI was dominant over femoral access PCI. Sensitivity analyses supported the robustness of these findings.

**Conclusions:**

Radial access is associated with improved patient outcomes and considerably lower costs relative to femoral access PCI. Our findings support radial access being the preferred approach for PCI across a variety of indications in Australia.

## INTRODUCTION

International guidelines support percutaneous coronary intervention (PCI) as the preferred means for coronary revascularization in the setting of acute coronary syndromes (ACS).^1^ In contemporary practice, access to the coronary arteries in PCI is achieved via the femoral or radial arteries. Although femoral access was traditionally favored for the ease of cannulation and direct access to the coronary arteries, there is considerable evidence of the benefits of radial access PCI. A recent Cochrane review found that relative to femoral access, radial access is associated with significant reductions in patient mortality, bleeding, and access‐site complications.^1^ This is supported by data from cardiac registries in the United States, United Kingdom, and Australia.^2^, ^3^, ^4^, ^5^ In addition to the greater safety profile of radial access, hospital length of stay (LOS) is considerably shorter relative to transfemoral PCI.^6^, ^7^

Despite an accumulating body of evidence pertaining to the comparative benefits of radial access, the uptake of transradial access PCI has been variable.^7^, ^8^ This may be attributed to patient factors, as well as operator preference and experience.^1^, ^2^, ^7^ Studies have highlighted the potential cost savings and cost‐effectiveness attributed to radial access PCI, including a recent Australian analysis of published data by our group.^3^, ^6^, ^7^, ^9^ Evidence from the Victorian Cardiac Outcomes Registry (VCOR) demonstrated that radial access is associated with improved patient outcomes and shorter LOS in Victoria, Australia, in line with international findings.^4^, ^8^ Furthermore, there has been considerable uptake in the number of radial access procedures over time, with the proportion of radial access PCIs overtaking femoral access in 2016.^4^, ^8^ Importantly, at present, a substantial proportion of PCIs are performed via transfemoral access and significant discrepancies persist in the uptake of radial access PCI across Victorian hospitals.^4^ In this context, we performed a cost‐effectiveness analysis of radial access PCI using data from VCOR to explore the health and economic benefits of radial access PCI.

## METHODS

### Data source

VCOR is a state‐wide cardiac clinical quality registry in Victoria, established in 2012 for the purposes of monitoring and benchmarking hospital performance and outcomes post‐PCI. ^10^ State‐wide coverage was achieved in 2017, and all public and private PCI‐capable centers currently participate and contribute data to VCOR.^10^, ^11^ Hospital‐appointed data managers collect data pertaining to patient characteristics at baseline, demographic characteristics, and procedural outcomes. Additional patient follow‐up is performed at 30 days for data on key patient outcomes, including mortality and major adverse cardiac and cerebrovascular events (MACCE), a composite of death, myocardial infarction, stroke, and target vessel revascularization. Additional details on VCOR have been described elsewhere.^10^, ^11^

The primary VCOR data set was linked to the Victorian Admitted Episodes Data set (VAED) to estimate the costs of PCIs.^12^, ^13^ The VAED contains admissions, diagnostic and procedural data across all Victorian hospitals; variables in the VAED reflect hospital activity for funding purposes.^12^, ^13^ Linkage with the National Death Index (NDI) was also performed to allow the estimation of long‐term mortality for patients undergoing PCI.^10^, ^12^ For the purposes of the present study, data on all consecutive PCI procedures in VCOR conducted over a 4‐year period between January 1, 2014, and December 31, 2017, were considered. The population was stratified by sex and indication for PCI (non‐ACS, unstable angina [UA], non‐ST‐elevation myocardial infarction [NSTEMI], and ST‐elevation myocardial infarction [STEMI]).

### Statistical analyses

Continuous variables were expressed as mean (standard deviation [SD]) or median (interquartile range [IQR]) where relevant, while categorical variables were expressed as frequencies (percentages). Pearson's *χ* ^2^ tests for categorical variables, and univariable linear regression modeling for continuous variables, were used to explore differences in patient and procedural characteristics between radial and femoral treatment arms. Generalized linear regression modeling (GLM) was used to overcome the high positive skew associated with patient LOS and door‐to‐balloon/device time parameters.^14^

Propensity score analyses were undertaken to reduce confounding arising from differences in characteristics of patients undergoing radial versus femoral PCIs. Additionally, the patient risk profile for PCI in Victoria has evolved, with patients presenting with greater risk over time.^9^ Inverse probability weighting (IPW) was used to construct a synthetic cohort in which the distribution of patient and procedural characteristics at baseline was independent of treatment assignment.^15^ Any bias attributed to differences in patient characteristics between the radial or femoral groups on key outcomes was therefore minimized.^15^ The following variables were used in predicting the use of radial access: age (<75 years and ≥75 years); sex; indigenous status; body mass index (BMI); in‐hours hospital arrival (between 08:00 and 18:00 on a workday); ACS category (non‐ACS, UA, NSTEMI, STEMI); cardiogenic shock or out‐of‐hospital cardiac arrest (OHCA) requiring intubation; medicated diabetes mellitus; peripheral vascular disease; cerebrovascular disease; chronic oral anticoagulation therapy; prior coronary artery bypass grafting; previous PCI; use of glycoprotein IIb/IIIa inhibitors; use of thienopyridine or ticagrelor; estimated glomerular filtration rate (eGFR); required mechanical ventricular support; lesion complexity (American College of Cardiology/American Heart Association Type A/B1 vs. Type B2/C lesions); unprotected left main PCI and in‐stent restenosis PCI. Balance on baseline covariates was evaluated using absolute standardized differences, with a value <10% considered as acceptable standardized bias.^16^ Propensity score weights were trimmed at the 5th and 95th percentiles to account for the effect of outlier weights in the model.^15^ The propensity score distribution for the IPW model is presented in Supporting Information, Appendix A.

Univariable logistic regression analyses were performed following IPW‐matching to explore differences in the incidence of patient clinical outcomes between radial and femoral groups. These are presented in Supporting Information, Appendix B.

## ECONOMIC EVALUATION

An economic model was developed to simulate the clinical and cost outcomes of radial versus femoral access for a hypothetical sample of 1000 individuals profiled on the IPW‐matched cohort.

## EFFECTIVENESS

Key outcomes considered were all‐cause mortality at 0–30 days and 31 days to 1 year, and major nonfatal bleeding at 0–30 days following index PCI. These outcomes were selected as the clinical benefit attributed to radial access are reductions in major bleeding and mortality events within the 12‐month period following PCI. The incidence of Bleeding Academic Research Consortium (BARC) Type 3 bleeding in the propensity‐matched population was used to inform the rate of nonfatal major bleeding events. Similarly, the incidence of all‐cause mortality at 30 days, and from 31 days to 1 year was informed by the incidence of all‐cause mortality in the propensity‐matched population for the relevant period. The incidence of key outcomes considered in the economic model is presented in Table 1.

**Table 1:** Inputs used in the economic model

Input	Sex	ACS subtype	Treatment arm		Distribution	Reference
			Radial	Femoral		
*Outcomes* a						
Point value (% *n*, range)						
Major bleed	Male^b^	Non‐ACS	0.29% (±15%)	0.66% (±15%)	Beta	VCOR IPW
		STEMI	1.37% (±15%)	2.70% (±15%)		
		NSTEMI	0.77% (±15%)	1.22% (±15%)		
		UA	0.77% (±15%)	0.56% (±15%)		
Mortality (0–30 days)	Male^b^	Non‐ACS	0.18% (±15%)	0.58% (±15%)		
		STEMI	2.90% (±15%)	6.88% (±15%)		
		NSTEMI	0.72% (±15%)	1.76% (±15%)		
		UA	0.36% (±15%)	0.60% (±15%)		
Mortality (31 days to 1 year)	Male^b^	Non‐ACS	0.95% (±15%)	1.75% (±15%)		
		STEMI	1.38% (±15%)	2.09% (±15%)		
		NSTEMI	1.70% (±15%)	2.08% (±15%)		
		UA	1.28% (±15%)	2.24% (±15%)		
Major bleed	Female^c^	Non‐ACS	0.55% (±15%)	1.33% (±15%)		
		STEMI	2.10% (±15%)	2.76% (±15%)		
		NSTEMI	0.63% (±15%)	1.36% (±15%)		
		UA	1.45% (±15%)	0.44% (±15%)		
Mortality (0–30 days)	Female^c^	Non‐ACS	0.39% (±15%)	0.79% (±15%)		
		STEMI	2.65% (±15%)	8.70% (±15%)		
		NSTEMI	0.94% (±15%)	1.33% (±15%)		
		UA	1.09% (±15%)	1.16% (±15%)		
Mortality (31 days to 1 year)	Female^c^	Non‐ACS	1.00% (±15%)	1.52% (±15%)		
		STEMI	1.54% (±15%)	4.02% (±15%)		
		NSTEMI	2.07% (±15%)	1.45% (±15%)		
		UA	0.34% (±15%)	1.16% (±15%)		
Cost variables						
Procedural cost^d^ (mean, 95% CI)	Male	Non‐ACS	$9816 ($9599–$10 032)	$10,816 ($10 344–$11,288)	Uniform	AR‐DRG^17^
		STEMI	$14 697 ($14 117–$15 277)	$17 246 ($16 481 – $18 011)		
		NSTEMI	$12 050 ($11 747–12 353)	$12 976 ($12 509–$13 443)		
		UA	$9364 ($8959–$9769)	$10 076 ($9418–$10 734)		
	Female	Non‐ACS	$9668 ($9389–$9946)	$10 921 ($10 388–$11 455)		
		STEMI	$14 276 ($13 537–$15 015)	$16 304 ($15 225–$17 384)		
		NSTEMI	$12 475 ($11 922–$13 028)	$12 983 ($12 601–$13 366)		
		UA	$9722 ($9108–$10 337)	$9893 ($9226–$10 561)		
Cost of bleeding (point value, ±25%)			$4585.15 (±25%)			
Cost of mortality (point value, ±25%)			$2131.73 (±25%)			
Utilities/disutilities						
Alive following PCI						
Non‐ACS (mean, 95% CI)			0.910 (0.900–0.910)		Beta	McCaffrey^18^
ACS (mean, 95% CI)			0.800 (0.789–0.811)		Beta	Lewis^19^
Bleeding (point value, ±15%)			−0.030 (±15%)		Log‐normal	Doble^20^

Abbreviations: ACS, acute coronary syndromes; PCI, percutaneous coronary intervention; NSTEMI, non‐ST‐elevation myocardial infarction; STEMI, ST‐elevation myocardial infarction; UA, unstable angina.

^a^ Based on incidence in IPW‐matched subgroups (*N* = 28 982).

^b^ The number of people included in the analyses of outcomes for males undergoing radial access PCI were non‐ACS, *n* = 5816, STEMI, *n* = 2396, NSTEMI, *n* = 3134, UA, *n* = 900; and males undergoing femoral access PCI were non‐ACS, *n* = 5466, STEMI, *n* = 1869, NSTEMI, *n* = 1883 and UA, *n* = 664.

^c^ The number of people included in the analyses of outcomes for females undergoing radial access PCI were non‐ACS, *n* = 1516, STEMI, *n* = 686, NSTEMI, *n* = 944, UA, *n* = 298; and females undergoing femoral access PCI were non‐ACS, *n* = 1,719; STEMI, *n* = 614; NSTEMI, *n* = 764, and UA, *n* = 313.

^d^ Based on generalized linear regression.

The economic model estimated the number of major nonfatal bleeding events, all‐cause‐mortality, quality‐adjusted life years (QALYs), years of life lived, and total costs. Costs were assessed from the perspective of the Australian public healthcare system. All costs were expressed in 2021 Australian dollars (AUD$).

## UTILITY VALUES

The utility values considered in the economic model are presented in Table 1.

Patients undergoing PCI for non‐ACS indications were assigned a utility of 0.91 (95% confidence interval [CI]: 0.90–0.91).^18^ Patients who underwent PCI for ACS indications were assigned a utility of 0.80 (95% CI: 0.79–0.81) to reflect that they had an initial ACS event. This utility value was derived from the estimated utility of patients following an ACS event in the Valsartan in Acute Myocardial Infarction (VALIANT) trial.^19^ A disutility of −0.03 was applied to patients who experienced a major bleeding event, in line with a recent study by Doble et al.^20^ This disutility was only applied to the initial 30‐day period, as major bleeding was considered an acute event occurring within the initial 30 days from the index procedure.

## COSTS

Key cost inputs used in the economic model are presented in Table 1.

## PROCEDURAL COSTS

Procedural costs were estimated using the Casemix funding method, in which a “weighted inlier‐equivalent separation” (WIES) weight is assigned to each episode of care.^21^, ^22^ The WIES reflects the cost of an episode of care relative to the average cost across all episodes of care and is multiplied by the WIES price set for a given financial year to estimate the cost for an episode of care. Each WIES weight was converted to dollar payments through the application of public sector payment rates for a given financial year to estimate the procedural cost of PCI.^23^ Univariable GLM with gamma distribution and log‐link was performed using the IPW‐matched population to estimate the average procedural costs associated with PCIs. Costs were stratified by sex and indication for PCI in the GLM. All costs were adjusted for inflation to 2021 AUD$ based upon the Health Price Index (HPI).^24^

## COST OF ACUTE EVENTS

Costs associated with procedural complications, including bleeding and mortality, were captured in the procedural costs. In estimating the cost of acute events occurring outside of the procedure and up to 1 year, Australian Refined‐Diagnosis Related Groups (AR‐DRG) data for hospitalizations were used.^17^ In lieu of available data on the costs of major bleeding due to PCI, the cost of a major bleeding event was assumed to be equivalent to the cost of gastrointestinal hemorrhage, in line with other studies which have considered the costs of major bleeding in the setting of cardiovascular disease in Australia.^25^, ^26^, ^27^

The cost of patient death from all causes occurring outside of the acute period (31 days to 1 year) was based on the weighted average of AR‐DRG codes F60B (Circulatory Disorders, Admitted for AMI Without Invasive Cardiac Investigation Procedure, Transferred <5 Days) and B70D (Stroke and Other Cerebrovascular Disorders, Transferred <5 Days). This is as mortality in the initial year following PCI is attributed to cardiovascular causes.^28^ It was conservatively assumed that hospitalization would only occur in only 50% of deaths. Therefore, only 50% of all deaths occurring outside of the initial hospital stay would incur hospitalizations costs, in line with previous studies.^25^, ^27^

## MODEL OUTCOMES

The main outcome of interest for the cost‐effectiveness analysis was the incremental cost‐effectiveness ratio (ICER) in terms of cost per QALY gained and cost per year of life saved (YoLS) for radial access compared with femoral access PCI.

## SENSITIVITY ANALYSES

A series of deterministic and probabilistic sensitivity analyses (PSA) was performed to explore the degree of uncertainty around key model parameters.^29^ Key input parameters were varied by the upper and lower limit of the 95% CI, or by ±15% or ±25% around the estimate (Table 1). Additional sensitivity analyses involved stratifying the radial and femoral groups by year of procedure, and the exclusion of high‐risk patient subgroups.^4^, ^30^ These include patients with high‐risk conditions requiring mechanical ventricular support or presenting with OHCA requiring intubation, patients aged >80 years, patients with an eGFR <90 ml/min/1.73 m^2^, and patients with Type B2/C lesion complexity.^4^, ^30^ Univariable logistic regression analyses were performed following IPW‐matching to explore differences in the incidence of patient clinical outcomes between radial and femoral groups for these high‐risk patient subgroups (Supporting Information, Appendix C). For the PSA, a second‐order Monte Carlo simulation with 10 000 iterations was performed using the ranges and probability distributions presented in Table 1.

## RESULTS

### Baseline characteristics

Baseline and procedural characteristics of the unadjusted VCOR population are presented in Table 2.

**Table 2:** Baseline and procedural characteristics of the VCOR and IPW‐matched cohorts

Variable	Unadjusted population			IPW‐matched cohort		
	Radial (*N* = 16 278)	Femoral (*N* = 15 920)	*p* value	Radial (*N* = 15 793)	Femoral (*N* = 13 189)	Total (*N* = 28 982)
*Baseline characteristics*						
Age (years)			<0.001			
Mean (SD)	64.1 (11.6)	67.1 (11.5)		63.7 (11.3)	65.7 (11.4)	64.6 (11.4)
Median (IQR)	64.0 (56.0, 73.0)	68.0 (59.0, 76.0)		64.0 (56.0, 72.0)	66.0 (58.0, 74.0)	65.0 (57.0, 73.0)
Age group (*n*, %*N*)						
<75	12 981 (79.8%)	11 308 (71.0%)		12 863 (81.5%)	10 020 (76.0%)	22 883 (79.0%)
≥75	3297 (20.3%)	4612 (29.0%)		2930 (18.6%)	3169 (24.0%)	6099 (21.0%)
Aboriginal/Torres Strait Islander (*n*, %*N*)	82 (0.50%)	57 (0.36%)	0.046	69 (0.4%)	46 (0.4%)	115 (0.4%)
Sex			<0.001			
Male	12 900 (79.3%)	11 918 (74.9%)		12 608 (79.8%)	10 067 (76.3%)	22 675 (78.2%)
Female	3378 (20.8%)	4002 (25.1%)		3185 (20.2%)	3122 (23.7%)	6307 (21.8%)
BMI (kg/m^2^)			<0.001			
Underweight (<18.5 kg/m^2^)	89 (0.6%)	119 (0.8%)		83 (0.5%)	87 (0.7%)	170 (0.6%)
Normal (18.5–24.9 kg/m^2^)	3421 (21.0%)	3585 (22.5%)		3482 (22.1%)	3028 (23.0%)	6510 (22.5%)
Overweight (25–29.9 kg/m^2^)	6490 (39.9%)	6337 (39.8%)		6325 (40.1%)	5261 (39.9%)	11 586 (40.0%)
Obese (≥30 kg/m^2^)	6066 (37.3%)	5652 (35.5%)		5903 (37.4%)	4813 (36.5%)	10 716 (37.0%)
Missing	212 (1.3%)	227 (1.4%)		‐	‐	‐
ACS type			<0.001			
Non‐ACS	7248 (44.5%)	8551 (53.7%)		6925 (43.9%)	6761 (51.3%)	13 686 (47.2%)
UA	1178 (7.2%)	1192 (7.5%)		1213 (7.7%)	983 (7.5%)	2196 (7.6%)
NSTEMI	4303 (26.4%)	3229 (20.3%)		4457 (28.2%)	2876 (21.8%)	7334 (25.3%)
STEMI	3549 (21.8%)	2948 (18.5%)		3197 (20.2%)	2570 (19.5%)	5767 (19.9%)
Cardiogenic shock	216 (1.3%)	510 (3.2%)	<0.001	137 (0.9%)	259 (2.0%)	396 (1.4%)
OHCA requiring intubation	108 (0.7%)	255 (1.6%)	<0.001	76 (0.5%)	151 (1.1%)	226 (0.8%)
Pre‐procedure cardiac arrest	163 (1.0%)	318 (2.0%)	<0.001	136 (0.9%)	213 (1.6%)	349 (1.2%)
LVEF grade			<0.001			
Normal (≥50%)	8472 (52.1%)	7941 (49.9%)		12 098 (76.6%)	9784 (74.2%)	21 882 (75.5%)
Mild (45%–49%)	2381 (14.6%)	2493 (15.7%)		2248 (14.2%)	2034 (15.4%)	4282 (14.8%)
Moderate (35%–44%)	1132 (7.0%)	1212 (7.6%)		1057 (6.7%)	954 (7.2%)	2011 (6.9%)
Severe (<35%)	439 (2.7%)	602 (3.8%)		390 (2.5%)	417 (3.2%)	807 (2.8%)
Missing	3854 (23.7%)	3672 (23.1%)		‐	‐	‐
Medicated diabetes (*n*, %*N*)	3254 (20.0%)	3900 (24.5%)	<0.001	3076 (19.5%)	2833 (21.5%)	5908 (20.4%)
Peripheral vascular disease	426 (2.6%)	734 (4.6%)	<0.001	358 (2.3%)	421 (3.2%)	778 (2.7%)
Cerebrovascular disease	481 (3.0%)	697 (4.4%)	<0.001	431 (2.7%)	461 (3.5%)	892 (3.1%)
Chronic oral anticoagulant therapy	837 (5.1%)	1023 (6.4%)	<0.001	761 (4.8%)	765 (5.8%)	1527 (5.3%)
Previous CABG	373 (2.3%)	2223 (14.0%)	<0.001	70 (0.4%)	343 (2.6%)	413 (1.4%)
Previous PCI	5033 (30.9%)	6419 (40.3%)	<0.001	4662 (29.5%)	4719 (35.8%)	9381 (32.4%)
Dialysis	83 (0.5%)	296 (1.9%)	<0.001	70 (0.4%)	161 (1.2%)	231 (0.8%)
Renal transplant	14 (0.1%)	82 (0.5%)	<0.001	12 (0.1%)	66 (0.5%)	78 (0.3%)
Renal replacement therapy			<0.001			
Yes	3 (0.0%)	15 (0.1%)		2 (0.0%)	8 (0.1%)	10 (0.0%)
Missing	85 (0.5%)	298 (1.9%)		‐	‐	‐
Fibrinolytic therapy	669 (4.1%)	293 (1.8%)	<0.001	615 (3.9%)	280 (2.1%)	895 (3.1%)
eGFR			<0.001			
Mean (SD)	97.0 (38.0)	86.0 (37.1)		98.3 (37.0)	91.0 (36.5)	94.9 (37.0)
Median (IQR)	92.8 (70.6, 117.8)	82.0 (60.1, 107.4)		94.1 (72.9, 118.5)	86.8 (66.4, 111.7)	90.9 (69.7, 115.5)
eGFR group			<0.001			
Normal (≥90 ml/min/1.73 m^2^)	12 721 (78.2%)	11 159 (70.1%)		13 944 (88.3%)	10 939 (82.9%)	24 884 (85.9%)
Moderate (60–89 ml/min/1.73 m^2^)	2107 (12.9%)	3103 (19.5%)		1748 (11.1%)	1956 (14.8%)	3705 (12.8%)
Severe (<30 ml/min/1.73 m^2^)	151 (0.9%)	588 (3.7%)		100 (0.6%)	293 (2.2%)	394 (1.4%)
Missing	1299 (8.0%)	1070 (6.7%)		‐	‐	‐
*Procedural characteristics*						
Peri‐procedural medications (*n*, %*N*)						
Glycoprotein IIb/IIIa inhibitor	1394 (8.6%)	1879 (11.8%)	<0.001	1231 (7.8%)	1439 (10.9%)	2670 (9.2%)
Thienopyridine or ticagrelor	13 061 (80.2%)	12 864 (80.8%)	0.199	12 630 (80%)	10 603 (80.4%)	23 234 (80.2%)
Aspirin	15 051 (92.7%)	13 827 (87.2%)	<0.001	14 601 (92.7%)	11 459 (87.2%)	26,060 (90.2%)
Antithrombin	14 701 (91.3%)	14 012 (88.9%)	<0.001	14 245 (91.2%)	11 686 (89.5%)	25 931 (90.4%)
Lesion characteristics (*n*, %*N*)						
Treated vessel			<0.001			
Left main coronary artery	154 (1.0%)	419 (2.6%)	<0.001	90 (0.6%)	175 (1.3%)	265 (0.9%)
Multilesion disease	3161 (19.4%)	3386 (21.3%)	<0.001	3045 (19.3%)	2805 (21.3%)	5850 (20.2%)
Multivessel disease	1043 (6.4%)	1208 (7.6%)	<0.001	973 (6.2%)	870 (6.6%)	1843 (6.4%)
Lesion complexity						
Type A or B1	7936 (48.8%)	5786 (36.2%)	<0.001	7988 (51%)	5519 (41.8%)	13 506 (46.6%)
Type B2 or C	8342 (51.3%)	10 152 (63.8%)		7805 (49.4%)	7670 (58.2%)	15 476 (53.4%)
Unprotected left main PCI (*n*, %*N*)	115 (0.7%)	232 (1.5%)	<0.001	82 (0.5%)	137 (1.0%)	219 (0.8%)
Chronic total occlusion (*n*, %*N*)	462 (2.8%)	862 (5.4%)	<0.001	419 (2.7%)	670 (5.1%)	1089 (3.8%)
In‐stent restenosis (*n*, %*N*)	787 (4.8%)	1188 (7.5%)	<0.001	704 (4.5%)	767 (5.8%)	1471 (5.1%)
Device used (*n*, %*N*)						
BMS only	1586 (89.7%)	1769 (11.1%)	<0.001	1566 (9.9%)	1505 (11.41%)	3072 (10.6%)
DES	13 753 (84.5%)	12 787 (80.3%)	<0.001	13 411 (84.9%)	10 780 (81.9%)	24 211 (83.5%)
POBA only	817 (5.0%)	1310 (8.2%)	<0.001	694 (4.4%)	834 (6.3%)	1528 (5.3%)
Intravenous ultrasound	175 (1.1%)	226 (1.4%)	0.005	168 (1.1%)	173 (1.3%)	341 (1.2%)

*Note*: There were 1 missing case for medicated diabetes status, 4 for out‐of‐hospital cardiac arrest, 1 for in‐hospital pre‐procedure cardiac arrest, 3 for peripheral vascular disease, 2 for cerebrovascular disease or chronic oral anticoagulant therapy and 1 for renal transplant.

Abbreviations: ACS, acute coronary syndrome; BMI, body mass index; BMS, bare metal stent; CABG, coronary artery bypass graft; DES, drug‐eluting stent; eGFR, estimated glomerular filtration rate; IPW, inverse probability weighted; LVEF, left ventricular ejection fraction; NSTEMI, non‐ST‐elevation myocardial infarction; OHCA, out‐of‐hospital cardiac arrest; POBA, plain old balloon angioplasty; STEMI, ST‐elevation myocardial infarction; UA, unstable angina.

Data from 32 198 patients undergoing PCI over the 4‐year period from January 1, 2014, to December 31, 2017, were analyzed. Of these, 16 278 (50.56%) involved radial access and 15 920 (49.44%) femoral access. Patient baseline characteristics were significantly different between radial and femoral groups. Compared with femoral access PCI, patients undergoing radial access PCI were younger (64 ± 12 vs. 67 ± 12 years), more likely to be male, and had a higher BMI. Furthermore, patients undergoing radial access were more likely to have an ACS indication for PCI, less likely to have a cardiogenic shock or cardiac arrest, and less likely to have the following comorbidities: diabetes mellitus, peripheral vascular disease, cerebrovascular disease, prior CABG or PCI and renal impairment.

## IPW ANALYSIS

**Figure 1:**
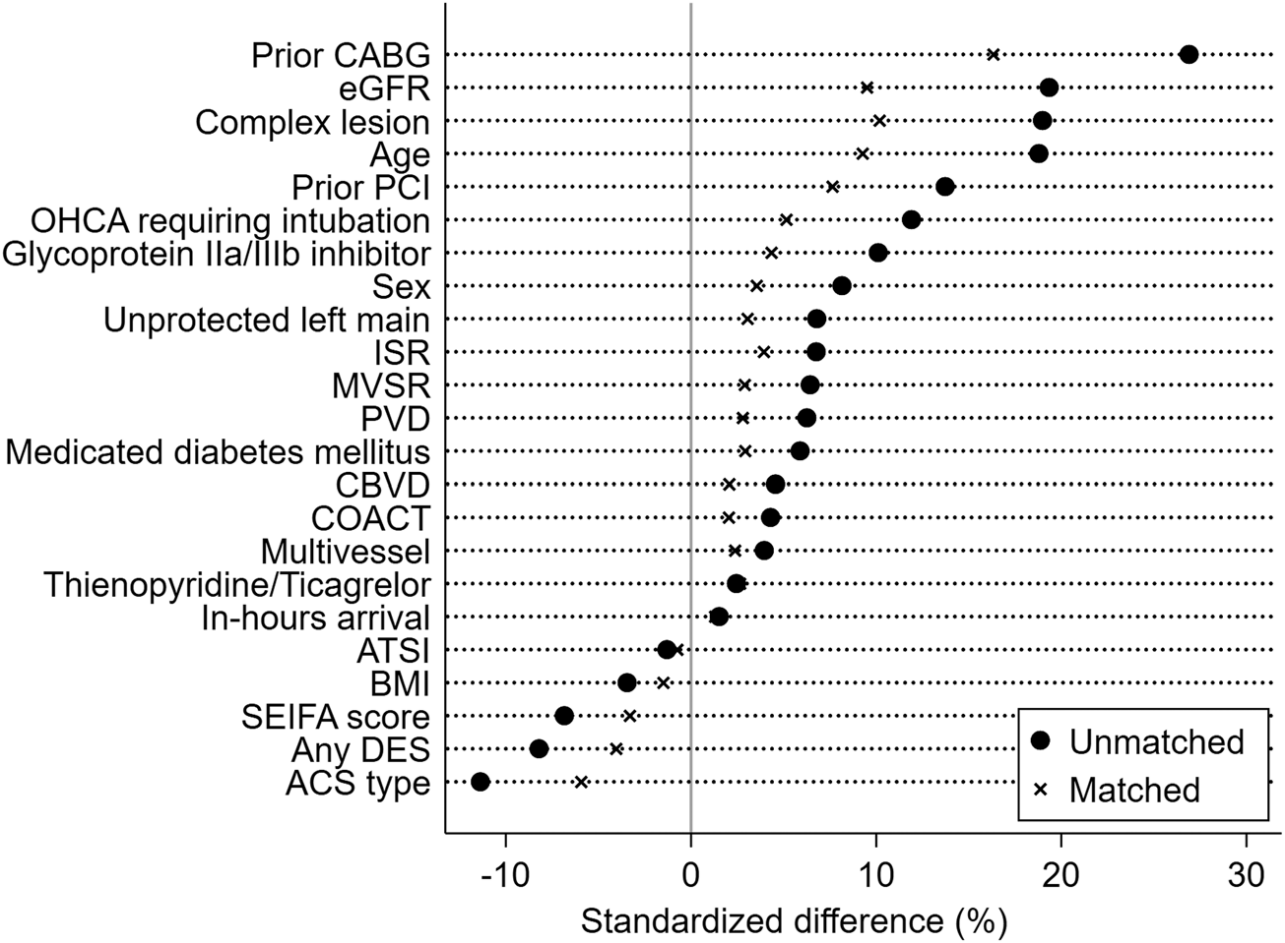
Standardised difference plot. ACS, acute coronary syndrome; ATSI, Aboriginal or Torres Strait Islander; BMI, body mass index; CABG, coronary artery bypass graft; CBVD, cerebrovascular disease; COACT, chronic oral anticoagulant therapy; eGFR, estimated glomerular filtration rate; ISR, in‐stent restenosis; MVSR, mechanical ventricular support; OHCA, out‐of‐hospital cardiac arrest; SEIFA, Socioeconomic Indexes for Areas

Figure 1 presents a plot of standardized mean differences before and after IPW‐matching.

IPW‐matched cohorts were similar across key variables of interest, with an absolute standardized difference <10% achieved for most variables. Baseline and procedural characteristics of the IPW‐matched cohorts are summarized in Table 2.

A total of 28 982 patients were included in the propensity score‐matched analysis, 15 793 in the radial group and 13 189 in the femoral group. However, differences in the proportion of patients with non‐ACS, shock, and OHCA remained. At least 83% of patients were retained following IPW‐matching and stratification of the radial and femoral groups into sex (male or female) and ACS subgroups (non‐ACS, UA, NSTEMI, STEMI).

## COST‐EFFECTIVENESS ANALYSES

The incidence of nonfatal major bleeding and all‐cause mortality in each of the radial and femoral groups in the IPW‐matched population was used to inform the model (see Table 1). The proportional distribution of ACS categories in the femoral PCI group was applied to the radial group. For males, the distribution of ACS categories was 55% non‐ACS, 7% UA, 19% NSTEMI, and 19% STEMI after IPW‐matching. For females, the proportional distribution across ACS categories was 50% non‐ACS, 9% UA, 22% NSTEMI, and 18% STEMI.

The incremental costs, clinical parameters, and ICERs from the base‐case analysis are summarized in Table 3. The incremental costs, clinical parameters, and ICERs from the analyses excluding high‐risk patients are presented in Supporting Information, Appendix D.

**Table 3:** Results of the base‐case analyses

Parameter	Treatment arm		Difference
	Radial access	Femoral access	
*Key acute clinical events*			
Nonfatal major bleeding	8	13	−6*
Mortality	21	40	−19
*Clinical effectiveness parameters*			
Total life years	986	971	15
Total QALYs	846	834	13*
*Cost parameters*			
Procedural costs	$11 132 434	$12 325 726	−$1 193 293
Acute events costs^a^			
Bleeding	$18 115	$21 225	−$3110
Mortality	$31 808	$50 093	−$18 286
Total costs	$11 182 357	$12 397 045	−$1 214 688
*Cost‐effectiveness parameters*	‐	‐	
Cost per YoLs			−$79 566
Cost per QALY gained			−$95 577

Abbreviations: QALY, quality‐adjusted life year; YoLs, year of life saved.

* Figures may not add up due to rounding.

^a^ Major bleeding and mortality occurring out of the index hospital stay.

For every 1000 patients undergoing PCI, radial access would prevent six nonfatal major bleeding episodes and 19 deaths over 1 year, compared to femoral access. The respective numbers needed to treat were 180 and 52. For every 1000 patients undergoing PCI, radial access would save 15 years of life and 13 QALYs in the year following the procedure, compared to femoral access, at a net cost saving of $1 214 688. Therefore, from a health economic point of view, radial access PCI was dominant over femoral access PCI.

## SENSITIVITY ANALYSES

The results of the one‐way, deterministic sensitivity analyses are presented in Table 4.

**Table 4:** Results of deterministic sensitivity analyses

Scenario	Difference		ICER
	Cost	QALY	
*Base case*	−$1 214 688	13	−$95 670
Time horizon (base case: 1 year)			
0–30 days	−$1 201 145	0	−$2 732 574
Incidence of outcomes			
Radial access			
Lower 15%	−$1 222 142	14	−$84 437
Upper 15%	−$1 207 246	11	−$110 539
Femoral access			
Lower 15%	−$1 204 147	9	−$133 258
Upper 15%	−$1 225 174	16	−$74 946
Utility inputs			
Initial health state (base case: 0.91 for non‐ACS, 0.80 for ACS)			
Lower limit (0.90 for non‐ACS, 0.80 for ACS)	−$1 214 688	13	−$96 924
Upper limit (0.91 for non‐ACS, 0.81 for ACS)	−$1 214 688	13	−$94 716
Bleeding disutility (base case: 0.03)			
Lower 15%	−$1 214 688	13	−$95 763
Upper 15%	−$1 214 688	13	−$95 577
Cost inputs			
Cost of bleeding			
Lower 25%	−$1 213 911	13	−$95 609
Upper 25%	−$1 215 466	13	−$95 731
Cost of mortality			
Lower 25%	−$1 210 117	13	−$95 310
Upper 25%	−$1 219 259	13	−$96 030
Radial procedure costs^a^			
Lower limit	−$1 598 627	13	−$125 909
Upper limit	−$830 750	13	−$65 431
Femoral procedure^a^			
Lower limit	−$640 057	13	−$50 411
Upper limit	−$1 789 316	13	−$140 928
Stratified by year (base case: 2014–2017)			
2014	−$1 239 826	11	−$108 346
2015	−$1 256 481	13	−$95 492
2016	−$1 221 746	18	−$68 829
2017	−$1 226 044	25	−$48 929
Exclusion of high‐risk subgroups (base case: all patients)			
OHCA/MVSR			
0–30 days	−$807 453	0	−$3 352 943
1 year	−$820 890	8	−$102 041
Aged > 80 years			
0–30 days	−$1 112 770	0	−$3 332 133
1 year	−$1 119 522	9	$125 300
eGFR < 90 ml/min/1.73 m^2^			
0–30 days	−$1 079 241	0	−$2 584 475
1 year	−$1 086 951	11	−$97 991
Type B2/C lesion complexity			
0–30 days	−$912 364	0	−$3 874 216
1 year	−$921 147	7	−$129 472

Abbreviations: eGFR, estimated glomerular filtration rate; ICER, incremental cost‐effectiveness ratio; OHCA, out‐of‐hospital cardiac arrest; MVSR, mechanical ventricular support required; QALY, quality‐adjusted life year.

^a^ Radial and femoral procedure costs were varied by the upper and lower limits of the 95% CI around the mean procedure cost estimated for each sex and ACS strata.

Based on the one‐way sensitivity analyses, the model was most sensitive to procedural costs and the year for which PCI occurred. However, the dominance of radial PCI over femoral PCI was maintained across the various scenario analyses. The results of the additional PSA are presented in Supporting Information, Appendix E. In the PSA, all of the iterations also fell within the dominant domain; that is, radial access was both health and cost‐saving relative to femoral access PCI.

## DISCUSSION

Our study has demonstrated that radial access is cost‐saving relative to femoral access PCI for patients across a variety of indications. Cost savings are attributable to reductions in hospital LOS and complications. Importantly, when exploring the impact of radial access over time in scenario analyses, the incremental costs attributed to radial access remained relatively stable while the incremental effectiveness increased. This suggests that further reductions in adverse patient outcomes and greater cost savings are likely to occur with improved uptake of radial access PCI and operator proficiency.^8^, ^31^ In a recently published study by our group, we explored the benefits of radial access using data from the Minimizing Adverse Haemorrhagic Events by Transradial Access Site and Systematic Implementation of Angiox (MATRIX) trial.^9^ Although this analysis was limited to exploring cost savings attributed to improved clinical outcomes for an ACS‐only population, we found that radial access was likely cost‐saving for the Australian health system. The limited number of studies exploring the cost‐effectiveness of radial access PCI also conclude that radial access is cost‐saving relative to femoral access PCI.^3^, ^7^, ^32^ Our current analyses using real‐world (VCOR) data captured the considerable cost savings attributable to reductions in patient LOS, in conjunction with improved clinical outcomes, across patients managed with PCI for a number of indications.

Importantly, our study also highlights the potential benefit of increasing the uptake of radial access for patients undergoing PCI for non‐ACS indications. In the VCOR IPW‐matched analyses, patients undergoing radial access PCI for STEMI and non‐ACS indications had a significantly lower incidence of major bleeding and all‐cause mortality relative to patients with femoral access. However, studies have predominantly explored the relative benefits of radial access PCI for the ACS population, with the greatest benefit observed for the high‐acuity STEMI population.^1^, ^6^, ^7^ Our analyses support expanding the use of radial access across a variety of indications for PCI, including non‐ACS PCIs, which contribute to a significant proportion of cases captured annually across Victoria and comprised 54% of the total VCOR cohort in the period of 2014–2017.^4^, ^11^, ^33^, ^34^, ^35^, ^36^ Notably, there are limited studies which explore the benefits attributed to radial access for a low‐acuity non‐ACS population, including patients undergoing PCI for elective reasons.^1^ The low uptake of radial access PCI among these patients is likely attributed to both the paucity of data in support of radial‐access PCI as well as operator preference for femoral access in patients in this population setting for PCI.^1^, ^31^

Furthermore, our base case results suggest that the mortality benefit attributed to radial access may be maintained over time. After IPW matching, the incidence of all‐cause mortality occurring at 31 days to 1 year was reduced amongst male non‐ACS patients (OR: 0.54, 95% CI: 0.39–0.74) (*p* < .001), and female STEMI patients (OR: 0.37, 95% CI: 0.18–0.76) (*p* = .007) treated through radial access PCI (Supporting Information, Appendix B). This persisted in subgroup analyses, excluding patients with OHCA/requiring mechanical ventricular support and for patients aged <80 years, but not for patients without complex (Type B2/C) lesions or patients with normal eGFR (Supporting Information, Appendix C). The additional mortality benefit captured beyond the acute period is likely attributed to a greater likelihood of intensive treatment with antithrombotic regimens following radial access PCI.^37^ Few studies have explored the potential long‐term efficacy of radial access PCI, but the evidence for mortality benefits attributed to radial access beyond the acute period following PCI is emerging.^1^, ^38^

A number of limitations to our study warrant mention. First, our data were drawn from an observational study (clinical registry), and although propensity score matching was undertaken to extract comparable groups, this strategy does not eliminate all sources of confounding.^6^, ^15^ In particular for our study, after IPW‐matching, patients in the femoral group still had higher risk profiles. However, the cost savings associated with radial access was maintained in scenario analyses excluding patients with OHCA requiring intubation or mechanical ventricular support, as well as other high‐risk factors (see Table 4 and Supporting Information, Appendix D).

Second, in the total VCOR cohort, 37% of patients were treated in private hospitals, but cost data were not available from private hospitals contributing data to VCOR. Hence, only unit costs from the public sector were applied. Public and private costs are likely to be different, due to differences in clinical characteristics between patients undergoing PCI in public and private hospitals, differences in patient management and differences in the relative efficiency between public and private hospitals.^4^, ^39^, ^40^ However, procedural costs were varied in sensitivity analyses, and the results remained consistent in terms of radial access being cost saving.

Third, the impact of access sites on the incidence of MACCE was not explicitly considered in our analyses. MACCE is less likely to occur with radial access,^7^ and hence we likely underestimated the cost‐savings attributed to radial access PCI. However, this would not have altered the conclusion of our study.

Finally, the time horizon of our evaluation was limited to the 12‐month period following index PCI. Although the evidence for the benefits of radial access lies within the short‐term period following PCI, additional longer‐term studies are warranted to examine additional longer‐term survival benefits and potential cost‐savings attributed to radial access.^1^

A key strength of our study lies in the large and contemporaneous cohort considered for IPW‐weighting. A similar analysis of patient outcomes using data captured between January 1, 2013, to December 31, 2014, in VCOR had identified significant reductions in major bleeding for patients with radial access PCI, but no association between access site selection and patient mortality at 30 days.^8^ In contrast, our analyses drew upon a larger data set spanning 4 years and better reflected the current literature in support of the benefits of radial access. Importantly, our analyses provide real‐world evidence for increasing the uptake of radial access PCI across Australia.

## CONCLUSIONS

Radial access PCI is cost saving and associated with significant clinical benefits relative to femoral access PCI in the Australian healthcare setting. Our findings support radial access being the preferred approach in PCI across a variety of indications.

## CONFLICT OF INTERESTS

P. L. is supported by an Australian Government Research Training Program (RTP) scholarship. E. Z. has received grants from Amgen, Astra Zeneca, Pfizer, Shire, and Zoll Medical Corporation outside of the submitted work. D. L. has received honoraria or study grants from Abbvie, Amgen, Astellas, AstraZeneca, Bohringer Ingelheim, Bristol Myers Squibb, Novartis, Pfizer, Sanofi, Shire, and Zoll Medical Corporation, outside the submitted work. D. S. is supported by the National Heart Foundation Fellowship and Viertel Foundation Award. C. R. is supported by a National Health and Medical Research Council Principal Research Fellowship (GNT1136372). The remaining authors declare that there are no conflict of interests.

## Supporting information

Supplementary Information

## Data Availability

All data are incorporated into the article and its online supplementary material.
