# Supplementary material for: The cost‐effectiveness of radial access percutaneous coronary intervention: A propensity‐score matched analysis of Victorian data
Source: Clin Cardiol. 2022 Feb 22;45(4):435–46. doi: 10.1002/clc.23798 (PMC9019896; doi:10.1002/clc.23798)
Supplement: Supplementary file 1 — Supplementary Information [file CLC-45-435-s001.docx]

**Appendix A**

**Figure A1: Distribution of IPW propensity scores by access site**


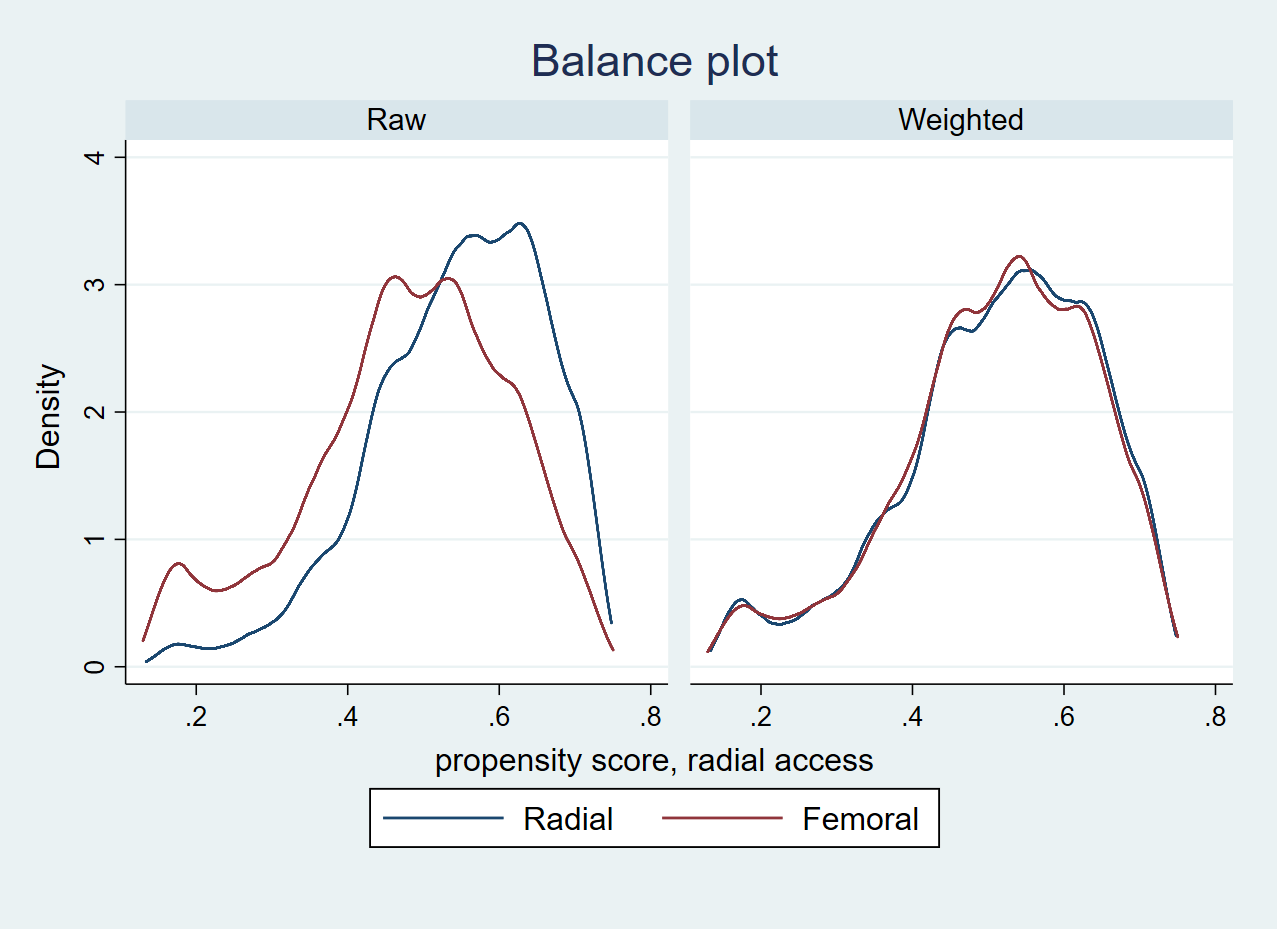


**Appendix B**

**Table B1: Incidence of key outcomes between radial and femoral groups following IPW-matching**

| **Input** | **Sex** | **ACS subtype** | **Treatment arm (%n)** | | **OR (95% CI)** | **P-value** |
| --- | --- | --- | --- | --- | --- | --- |
|  |  |  | **Radial** | **Femoral** |  |  |
| Major bleed | Male | Non-ACS | 0.29% | 0.66% | 0.45 (0.25 – 0.80) | 0.006 |
|  |  | STEMI | 1.37% | 2.70% | 0.51 (0.34 – 0.78) | 0.002 |
|  |  | NSTEMI | 0.77% | 1.22% | 0.65 (0.38 – 1.13) | 0.127 |
|  |  | UA | 0.77% | 0.56% | 1.38 (0.35 – 5.36) | 0.645 |
| Mortality (0-30d) |  | Non-ACS | 0.18% | 0.58% | 0.30 (0.16 – 0.57) | <0.001 |
|  |  | STEMI | 2.90% | 6.88% | 0.40 (0.31 – 0.53) | <0.001 |
|  |  | NSTEMI | 0.72% | 1.76% | 0.40 (0.24 – 0.66) | <0.001 |
|  |  | UA | 0.36% | 0.60% | 0.59 (0.14 – 2.38) | 0.455 |
| Mortality (31d – 1 yr) |  | Non-ACS | 0.95% | 1.75% | 0.54 (0.39 – 0.74) | <0.001 |
|  |  | STEMI | 1.38% | 2.09% | 0.66 (0.41 – 1.04) | 0.073 |
|  |  | NSTEMI | 1.70% | 2.08% | 0.82 (0.54 – 1.22) | 0.324 |
|  |  | UA | 1.28% | 2.24% | 0.56 (0.26 – 1.22) | 0.144 |
| Major bleed | Female | Non-ACS | 0.55% | 1.33% | 0.41 (0.19 – 0.92) | 0.030 |
|  |  | STEMI | 2.10% | 2.76% | 0.67 (0.35 – 1.29) | 0.234 |
|  |  | NSTEMI | 0.63% | 1.36% | 0.54 (0.22 – 1.35) | 0.190 |
|  |  | UA | 1.45% | 0.44% | 3.32 (0.60 – 18.34) | 0.169 |
| Mortality (0-30d) |  | Non-ACS | 0.39% | 0.79% | 0.49 (0.21 -1.16) | 0.106 |
|  |  | STEMI | 2.65% | 8.70% | 0.29 (0.18 – 0.46) | <0.001 |
|  |  | NSTEMI | 0.94% | 1.33% | 0.71 (0.30 – 1.66) | 0.429 |
|  |  | UA | 1.09% | 1.16% | 0.94 (0.21 – 4.25) | 0.936 |
| Mortality (31d – 1 yr) |  | Non-ACS | 1.00% | 1.52% | 0.65 (0.35 – 1.23) | 0.187 |
|  |  | STEMI | 1.54% | 4.02% | 0.37 (0.18 – 0.76) | 0.007 |
|  |  | NSTEMI | 2.07% | 1.45% | 1.43 (0.71 – 2.90) | 0.316 |
|  |  | UA | 0.34% | 1.16% | 0.29 (0.03 – 2.59) | 0.265 |

ACS= acute coronary syndrome; d = day; NSTEMI = non-ST-elevation myocardial infarction; STEMI = ST-elevation myocardial infarction; UA = unstable angina; yr = year

**Appendix C**

**Table C1: Incidence of key outcomes between radial and femoral groups following IPW-matching (excluding out-of-hospital cardiac arrest or patients requiring mechanical ventricular support)**

| **Input** | **Sex** | **ACS subtype** | **Treatment Arm** | | **OR (95% CI)** | **P-value** |
| --- | --- | --- | --- | --- | --- | --- |
|  |  |  | **Radial** | **Femoral** |  |  |
| Major bleed | Male | Non-ACS | *0.27%* | *0.62%* | 0.45 (0.24 ­ 0.82) | 0.009 |
|  |  | STEMI | *1.04%* | *2.04%* | 0.52 (0.31 ­ 0.88) | 0.014 |
|  |  | NSTEMI | *0.75%* | *1.19%* | 0.64 (0.36 ­ 1.12) | 0.115 |
|  |  | UA | *0.77%* | *0.56%* | 1.37 (0.35 ­ 5.32) | 0.652 |
| Mortality (0-30d) |  | Non-ACS | *0.12%* | *0.46%* | 0.25 (0.11 ­ 0.56) | 0.001 |
|  |  | STEMI | *1.58%* | *2.17%* | 0.72 (0.46 ­ 1.14) | 0.165 |
|  |  | NSTEMI | *0.49%* | *1.55%* | 0.32 (0.17 ­ 0.57) | <0.001 |
|  |  | UA | *0.35%* | *0.52%* | 0.68 (0.16 ­ 2.96) | 0.611 |
| Mortality (31d – 1 yr) |  | Non-ACS | *0.94%* | *1.72%* | 0.54 (0.39 ­ 0.75) | <0.001 |
|  |  | STEMI | *1.35%* | *1.98%* | 0.68 (0.41 ­ 1.11) | 0.123 |
|  |  | NSTEMI | *1.68%* | *2.09%* | 0.80 (0.53 ­ 1.20) | 0.283 |
|  |  | UA | *1.28%* | *2.25%* | 0.56 (0.26 ­ 1.21) | 0.143 |
| Major bleed | Female | Non-ACS | *0.55%* | *1.30%* | 0.42 (0.19 ­ 0.94) | 0.035 |
|  |  | STEMI | *1.91%* | *2.39%* | 0.75 (0.36 ­ 1.57) | 0.448 |
|  |  | NSTEMI | *0.63%* | *1.32%* | 0.56 (0.22 ­ 1.41) | 0.218 |
|  |  | UA | *1.47%* | *0.44%* | 3.36 (0.61 ­ 18.57) | 0.165 |
| Mortality (0-30d) |  | Non-ACS | *0.32%* | *0.79%* | 0.40 (0.16 ­ 1.03) | 0.056 |
|  |  | STEMI | *1.49%* | *4.88%* | 0.29 (0.15 ­ 0.58) | <0.001 |
|  |  | NSTEMI | *0.87%* | *1.15%* | 0.75 (0.30 ­ 1.89) | 0.544 |
|  |  | UA | *1.11%* | *1.16%* | 0.95 (0.21 ­ 4.30) | 0.950 |
| Mortality (31d – 1 yr) |  | Non-ACS | *1.00%* | *1.48%* | 0.67 (0.36 ­ 1.27) | 0.222 |
|  |  | STEMI | *1.52%* | *4.08%* | 0.36 (0.17 ­ 0.77) | 0.008 |
|  |  | NSTEMI | *1.97%* | *1.43%* | 1.39 (0.68 ­ 2.86) | 0.371 |
|  |  | UA | *0.33%* | *1.17%* | 0.28 (0.03 ­ 2.56) | 0.262 |

ACS= acute coronary syndrome; d = day; NSTEMI = non-ST-elevation myocardial infarction; STEMI = ST-elevation myocardial infarction; UA = unstable angina; yr = year

**Table C2: Incidence of key outcomes between radial and femoral groups following IPW-matching (excluding patients >80 years)**

| **Input** | **Sex** | **ACS subtype** | **Treatment Arm** | | **OR (95% CI)** | **P-value** |
| --- | --- | --- | --- | --- | --- | --- |
|  |  |  | **Radial** | **Femoral** |  |  |
| Major bleed | Male | Non-ACS | *0.29%* | *0.57%* | 0.51 (0.28 ­ 0.94) | 0.031 |
|  |  | STEMI | *1.20%* | *2.53%* | 0.48 (0.30 ­0.75) | 0.002 |
|  |  | NSTEMI | *0.72%* | *0.93%* | 0.79 (0.42 ­1.47) | 0.453 |
|  |  | UA | *0.57%* | *0.62%* | 0.92 (0.22 ­3.90) | 0.915 |
| Mortality (0-30d) |  | Non-ACS | *0.11%* | *0.49%* | 0.22 (0.09 ­ 0.51) | <0.001 |
|  |  | STEMI | *2.33%* | *5.98%* | 0.38 (0.28 ­ 0.51) | <0.001 |
|  |  | NSTEMI | *0.56%* | *1.12%* | 0.50 (0.27 ­ 0.93) | 0.028 |
|  |  | UA | *0.37%* | *0.32%* | 1.16 (0.23 ­ 5.91) | 0.859 |
| Mortality (31d – 1 yr) |  | Non-ACS | *0.75%* | *1.21%* | 0.62 (0.42 ­ 0.91) | 0.016 |
|  |  | STEMI | *1.00%* | *1.55%* | 0.64 (0.37 ­ 1.11) | 0.112 |
|  |  | NSTEMI | *1.39%* | *1.49%* | 0.93 (0.58 ­ 1.51) | 0.779 |
|  |  | UA | *0.73%* | *1.73%* | 0.42 (0.16 ­ 1.10) | 0.077 |
| Major bleed | Female | Non-ACS | *0.50%* | *1.20%* | 0.42 (0.17 ­ 1.05) | 0.062 |
|  |  | STEMI | *1.75%* | *2.38%* | 0.63 (0.29 ­ 1.38) | 0.248 |
|  |  | NSTEMI | *0.32%* | *1.84%* | 0.24 (0.07 ­ 0.80) | 0.020 |
|  |  | UA | *1.65%* | *0.00%* | - | - |
| Mortality (0-30d) |  | Non-ACS | *0.21%* | *0.50%* | 0.41 (0.12 ­ 1.37) | 0.150 |
|  |  | STEMI | *1.71%* | *6.34%* | 0.26 (0.14 ­ 0.48) | <0.001 |
|  |  | NSTEMI | *0.69%* | *0.92%* | 0.75 (0.24 ­ 2.35) | 0.619 |
|  |  | UA | *1.24%* | *0.00%* | - | - |
| Mortality (31d – 1 yr) |  | Non-ACS | *0.99%* | *0.89%* | 1.11 (0.53 ­ 2.33) | 0.776 |
|  |  | STEMI | *1.12%* | *2.99%* | 0.37 (0.15 ­ 0.90) | 0.028 |
|  |  | NSTEMI | *1.47%* | *0.98%* | 1.50 (0.61 ­ 3.68) | 0.376 |
|  |  | UA | *0.37%* | *1.04%* | 0.35 (0.04 ­ 3.43) | 0.367 |

ACS= acute coronary syndrome; d = day; NSTEMI = non-ST-elevation myocardial infarction; STEMI = ST-elevation myocardial infarction; UA = unstable angina; yr = year

**Table C3: Incidence of key outcomes between radial and femoral groups following IPW-matching (excluding patients with Type B2/C complex lesion)**

| **Input** | **Sex** | **ACS subtype** | **Treatment Arm** | | **OR (95% CI)** | **P-value** |
| --- | --- | --- | --- | --- | --- | --- |
|  |  |  | **Radial** | **Femoral** |  |  |
| Major bleed | Male | Non-ACS | *0.23%* | *0.57%* | 0.40 (0.15 ­1.04) | 0.061 |
|  |  | STEMI | *0.95%* | *3.98%* | 0.26 (0.12 ­ 0.56) | <0.001 |
|  |  | NSTEMI | *0.74%* | *1.03%* | 0.74 (0.31 ­ 1.78) | 0.503 |
|  |  | UA | *0.59%* | *1.25%* | 0.47 (0.09 ­2.35) | 0.356 |
| Mortality (0-30d) |  | Non-ACS | *0.06%* | *0.34%* | 0.18 (0.04 ­ 0.85) | 0.030 |
|  |  | STEMI | *1.57%* | *4.54%* | 0.33 (0.18 ­ 0.62) | <0.001 |
|  |  | NSTEMI | *0.53%* | *1.49%* | 0.35 (0.15 ­ 0.84) | 0.019 |
|  |  | UA | *0.45%* | *0.00%* | - | - |
| Mortality (31d – 1 yr) |  | Non-ACS | *0.73%* | *1.16%* | 0.62 (0.35 ­1.11) | 0.107 |
|  |  | STEMI | *1.01%* | *1.41%* | 0.71 (0.27 ­1.85) | 0.487 |
|  |  | NSTEMI | *1.72%* | *1.48%* | 1.17 (0.59 ­ 2.33) | 0.660 |
|  |  | UA | *0.54%* | *1.07%* | 0.52 (0.20 ­ 1.37) | 0.186 |
| Major bleed | Female | Non-ACS | *0.58%* | *0.69%* | 0.85 (0.25 ­ 2.83) | 0.787 |
|  |  | STEMI | *1.69%* | *2.50%* | 0.72 (0.23 ­ 2.20) | 0.561 |
|  |  | NSTEMI | *0.81%* | *1.35%* | 0.60 (0.14 ­ 2.45) | 0.473 |
|  |  | UA | *0.00%* | *0.00%* | - | - |
| Mortality (0-30d) |  | Non-ACS | *0.20%* | *0.09%* | 2.34 (0.21 ­ 25.90) | 0.489 |
|  |  | STEMI | *1.93%* | *4.48%* | 0.42 (0.16 ­ 1.09) | 0.074 |
|  |  | NSTEMI | *0.24%* | *0.36%* | 0.66 (0.59 ­ 7.36) | 0.736 |
|  |  | UA | *0.00%* | *2.37%* | - | - |
| Mortality (31d – 1 yr) |  | Non-ACS | *0.83%* | *1.72%* | 0.48 (0.19 ­ 1.20) | 0.115 |
|  |  | STEMI | *1.74%* | *3.13%* | 0.55 (0.17 ­ 1.80) | 0.321 |
|  |  | NSTEMI | *1.58%* | *0.60%* | 2.63 (0.55 ­ 12.69) | 0.226 |
|  |  | UA | *0.54%* | *1.07%* | 0.50 (0.04 ­ 5.74) | 0.580 |

ACS= acute coronary syndrome; d = day; NSTEMI = non-ST-elevation myocardial infarction; STEMI = ST-elevation myocardial infarction; UA = unstable angina; yr = year

**Table C4: Incidence of key outcomes between radial and femoral groups following IPW-matching (normal eGFR only)**

| **Input** | **Sex** | **ACS subtype** | **Treatment Arm** | | **OR (95% CI)** | **P-value** |
| --- | --- | --- | --- | --- | --- | --- |
|  |  |  | **Radial** | **Femoral** |  |  |
| Major bleed | Male | Non-ACS | *0.29%* | *0.69%* | 0.42 (0.24 ­ 0.75) | 0.003 |
|  |  | STEMI | *1.35%* | *2.66%* | 0.52 (0.34 ­ 0.79) | 0.002 |
|  |  | NSTEMI | *0.82%* | *1.21%* | 0.68 (0.39 ­ 1.19) | 0.176 |
|  |  | UA | *0.77%* | *0.57%* | 1.36 (0.35 ­ 5.30) | 0.656 |
| Mortality (0-30d) |  | Non-ACS | *0.17%* | *0.58%* | 0.29 (0.15 ­ 0.56) | <0.001 |
|  |  | STEMI | *2.65%* | *6.66%* | 0.38 (0.29 ­ 0.50) | <0.001 |
|  |  | NSTEMI | *0.69%* | *1.59%* | 0.43 (0.26 ­ 0.72) | 0.002 |
|  |  | UA | *0.36%* | *0.36%* | 0.98 (0.19 ­ 5.02) | 0.983 |
| Mortality (31d – 1 yr) |  | Non-ACS | *0.91%* | *1.57%* | 0.58 (0.41 ­ 0.81) | <0.001 |
|  |  | STEMI | *1.32%* | *1.95%* | 0.67 (0.42 ­ 1.08) | 0.100 |
|  |  | NSTEMI | *1.75%* | *1.95%* | 0.90 (0.59 ­ 1.36) | 0.608 |
|  |  | UA | *1.29%* | *2.09%* | 0.61 (0.28 ­ 1.35) | 0.222 |
| Major bleed | Female | Non-ACS | *0.50%* | *1.28%* | 0.39 (0.17 ­ 0.91) | 0.029 |
|  |  | STEMI | *1.92%* | *2.78%* | 0.63 (0.32 ­ 1.26) | 0.190 |
|  |  | NSTEMI | *0.51%* | *1.75%* | 0.35 (0.13 ­ 0.94) | 0.036 |
|  |  | UA | *1.47%* | *0.48%* | 3.09 (0.56 ­ 17.10) | 0.195 |
| Mortality (0-30d) |  | Non-ACS | *0.35%* | *0.70%* | 0.50 (0.20 ­ 1.26) | 0.142 |
|  |  | STEMI | *2.28%* | *8.14%* | 0.26 (0.16 ­ 0.45) | <0.001 |
|  |  | NSTEMI | *0.89%* | *1.09%* | 0.82 (0.32 ­ 2.13) | 0.687 |
|  |  | UA | *1.10%* | *1.19%* | 0.93 (0.20 ­ 4.20) | 0.923 |
| Mortality (31d – 1 yr) |  | Non-ACS | *0.96%* | *1.03%* | 0.93 (0.46 ­ 1.90) | 0.845 |
|  |  | STEMI | *1.70%* | *3.21%* | 0.52 (0.25 ­1.11) | 0.090 |
|  |  | NSTEMI | *1.57%* | *0.89%* | 1.78 (0.71 ­ 4.43) | 0.219 |
|  |  | UA | *0.36%* | *0.98%* | 0.37 (0.04 ­ 3.55) | 0.385 |

ACS= acute coronary syndrome; d = day; NSTEMI = non-ST-elevation myocardial infarction; STEMI = ST-elevation myocardial infarction; UA = unstable angina; yr = year

**Appendix D**

**Table D1: Results of the subgroup analyses excluding patients with out-of-hospital cardiac arrest or requiring mechanical ventricular support**

| **Parameter** | **Treatment arm** | | **Difference ^*^** |
| --- | --- | --- | --- |
|  | **Radial access** | **Femoral access** |  |
| Key acute clinical events  Non-fatal major bleeding  Mortality | 7  18 | 12  31 | -5  -13 |
| Clinical effectiveness parameters  Total life years  Total QALYs | 989  850 | 979  842 | 9  8 |
| Cost parameters  Procedural costs  Acute events costs ^†^  Bleeding  Mortality  Total costs | $10,798,312  $18,123  $30,818  $10,847,254 | $11,597,447  $21,355  $49,342  $11,668,144 | -$799,135  -$3,231  -$18,524  -$820,890 |
| Cost-effectiveness parameters  Cost per YoLs  Cost per QALY gained | - | - | -$86,770  -$102,041 |

QALY = quality-adjusted life year; YoLs = year of life saved

^*^ Figures may not add up due to rounding

^†^Major bleeding and mortality occurring out of the index hospital stay

**Table D2: Results of the subgroup analyses excluding patients aged > 80 years**

| **Parameter** | **Treatment arm** | | **Difference^*^** |
| --- | --- | --- | --- |
|  | **Radial access** | **Femoral access** |  |
| Key acute clinical events  Non-fatal major bleeding  Mortality | 7  16 | 12  29 | -6  -13 |
| Clinical effectiveness parameters  Total life years  Total QALYs | 989  849 | 978  840 | 11  9 |
| Cost parameters  Procedural costs  Acute events costs ^†^  Bleeding  Mortality  Total costs | $11,099,094  $17,017  $25,984  $11,142,095 | $12,207,667  $17,830  $36,119  $12,261,617 | -$1,108,573  -$813  $10,135  -$1,119,522 |
| Cost-effectiveness parameters  Cost per YoLs  Cost per QALY gained | -  - | -  - | -$103,988  -$125,300 |

QALY = quality-adjusted life year; YoLs = year of life saved

^*^ Figures may not add up due to rounding

^†^ Major bleeding and mortality occurring out of the index hospital stay

**Table D3: Results of the subgroup analyses excluding patients with estimated glomerular filtration rate < 90 ml/min/1.73m^2^**

| **Parameter** | **Treatment arm** | | **Difference ^*^** |
| --- | --- | --- | --- |
|  | **Radial access** | **Femoral access** |  |
| Key acute clinical events  Non-fatal major bleeding  Mortality | 7  20 | 14  36 | -6  -16 |
| Clinical effectiveness parameters  Total life years  Total QALYs | 987  847 | 973  836 | 13  11 |
| Cost parameters  Procedural costs  Acute events costs ^†^  Bleeding  Mortality  Total costs | $11,092,194  $18,473  $30,542  $11,141,209 | $12,163,878  $21,831  $42,451  $12,228,160 | -$1,071,684  -$3,358  -$11,909  -$1,086,951 |
| Cost-effectiveness parameters  Cost per YoLs  Cost per QALY gained | -  - | -  - | -$81,376  -$97,991 |

QALY = quality-adjusted life year; YoLs = year of life saved

^*^ Figures may not add up due to rounding

^†^Major bleeding and mortality occurring out of the index hospital stay

**Table D4: Results of the subgroup analyses excluding patients with Type B2/C complex lesions**

| **Parameter** | **Treatment arm** | | **Difference ^*^** |
| --- | --- | --- | --- |
|  | **Radial access** | **Femoral access** |  |
| Key acute clinical events  Non-fatal major bleeding  Mortality | 6  15 | 12  26 | -5  -11 |
| Clinical effectiveness parameters  Total life years  Total QALYs | 991  851 | 982  844 | 9  7 |
| Cost parameters  Procedural costs  Acute events costs ^†^  Bleeding  Mortality  Total costs | $10,750,914  $16,130  $25,407  $10,792,451 | $11,654,294  $21,571  $37,733  $11,713,598 | -$903,380  -$5,441  -$12,326  -$921,147 |
| Cost-effectiveness parameters  Cost per YoLs  Cost per QALY gained | -  - | -  - | -$108,288  -$129,472 |

QALY = quality-adjusted life year; YoLs = year of life saved

^*^ Figures may not add up due to rounding

^†^Major bleeding and mortality occurring out of the index hospital stay

**Appendix E**

**Figure E1: Results of the probabilistic sensitivity analysis**


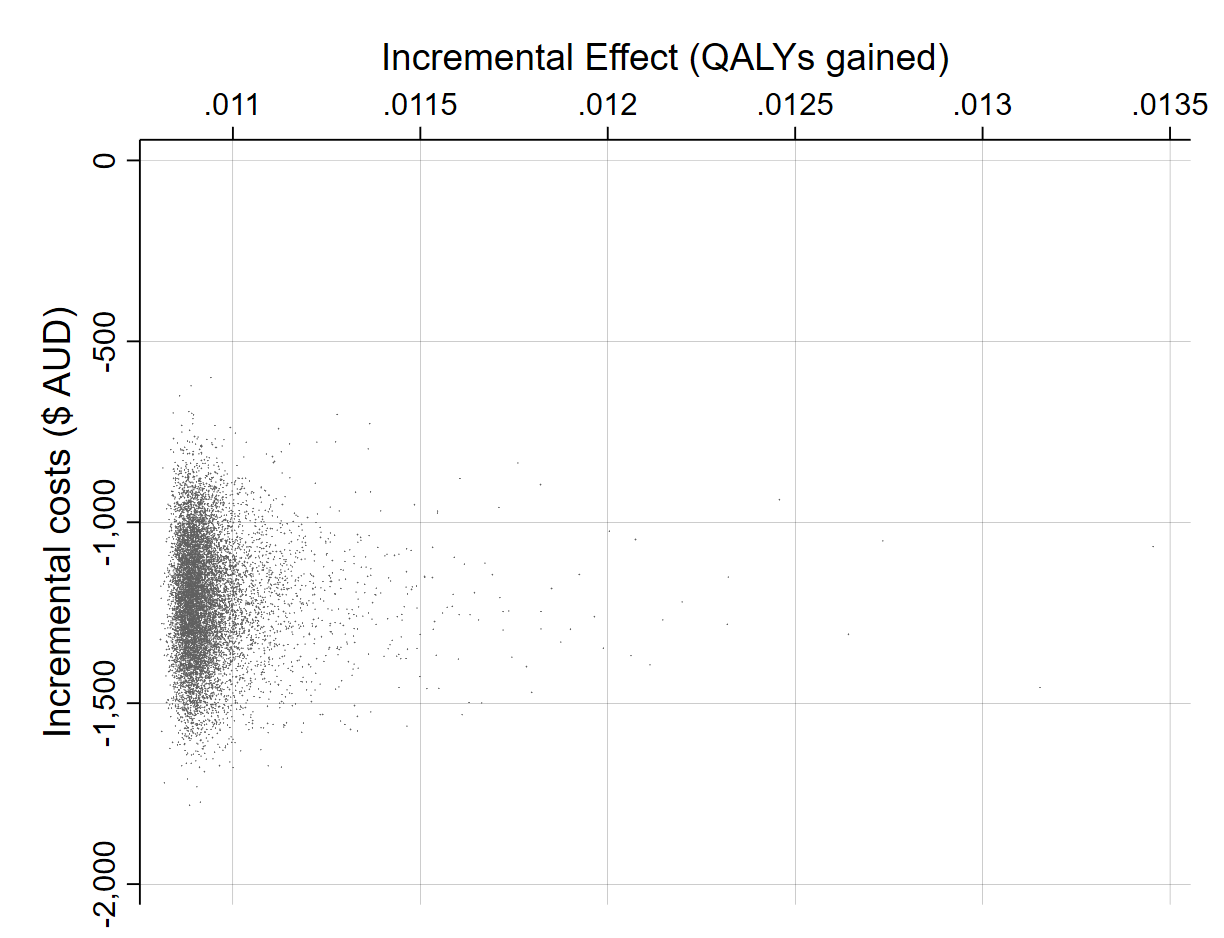


AUD = Australian dollars; QALY = quality-adjusted life year
